# Middle cerebral artery infarction, A rare complication of intracranial cryptococcoma in an immunocompetent patient: A case report and literature review

**DOI:** 10.3389/fsurg.2023.1083833

**Published:** 2023-02-15

**Authors:** Ying-Ching Li, Chun-Chia Tseng, Shuo-Chi Chien, Sheng-Han Huang, Tin-Wei Chang, Chun-Ting Chen, Po-Hsun Tu, Zhuo-Hao Liu, Yin-Cheng Huang

**Affiliations:** Department of Neurosurgery, Chang Gung Memorial Hospital, Linkou Branch, Taoyuan, Taiwan

**Keywords:** MCA infarction, cryptococcomas, rare complication, intracranial infection, immunocompetent adult infection

## Abstract

**Background:**

This report presents the first case of intracranial cryptococcoma arising from the right frontal lobe causing right middle cerebral artery infarction. Intracranial cryptococcomas usually occur in the cerebral parenchyma, basal ganglia, cerebellum, pons, thalamus, and choroid plexus; they may mimic intracranial tumors, but seldom cause infarction. ﻿Of the 15 cases of pathology-confirmed intracranial cryptococcomas in the literature, no case has been complicated by middle cerebral artery (MCA) infarction. Here, we discuss a case of intracranial cryptococcoma with an ipsilateral middle cerebral artery infarction.

**Case Description:**

A 40-year-old man was referred to our emergency room due to progressive headaches and acute left hemiplegia. The patient was a construction worker with no history of avian contact, recent travel, or human immunodeficiency virus (HIV) infection. Brain computed tomography (CT) showed an intra-axial mass, and subsequent magnetic resonance imaging (MRI) delineated a large mass of 53 mm in the right middle frontal lobe and a small lesion of 18 mm in the right caudate head, with marginal enhancement and central necrosis. A neurosurgeon was consulted in view of the intracranial lesion, and the patient underwent en-bloc excision of the solid mass. The pathology report later identified a *Cryptococcus* infection rather than malignancy. The patient underwent 4 weeks of postoperative treatment with amphotericin B plus flucytosine; he then received subsequent oral antifungal treatment for 6 months, and had neurologic sequelae that manifested as left side hemiplegia.

**Conclusion:**

Diagnosis of fungal infections in the CNS remains challenging. This is especially true of *Cryptococcus* CNS infections that present as a space-occupying lesion in an immunocompetent patient. A *Cryptococcus* infection should be considered in the differential diagnoses in patients with brain mass lesions, as this infection can be misdiagnosed as a brain tumor.

## Introduction

Diagnosis and treatment of central nervous system (CNS) cryptococcoma is more complicated than of meningitis. First, cryptococcomas are radiologically non-specific, and may be mistaken for malignant lesions; diagnosis may therefore only be made on histologic examination after resection. Second, the symptoms are unspecific. Common symptoms identified in our literature review, which included headaches, seizures and fever, are often seen in other CNS diseases as well. The common initial signs of cryptococcomas are an increasing intracranial pressure (IICP) and neurological focal signs. There has been no case in which hemiplegia was present according to our literature review. Of the 15 cases of pathology-confirmed intracranial cryptococcoma in the literature, there was no case complicated by middle cerebral artery (MCA) infarction. Here, we discuss a case of intracranial cryptococcoma with an ipsilateral MCA infarction.

## Clinical presentation

**Figure 1:**
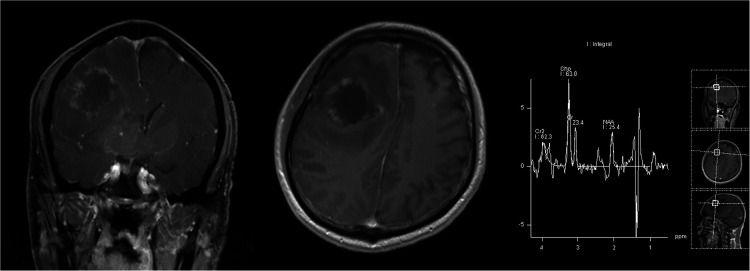
Post-contrast T1-weighted images showed a right frontal mass with a rugged marginal enhancement and possible extensive interior necrotic change; this was also suggested by the presence of a lactic acid peak on MR spectroscopy.

**Figure 2:**
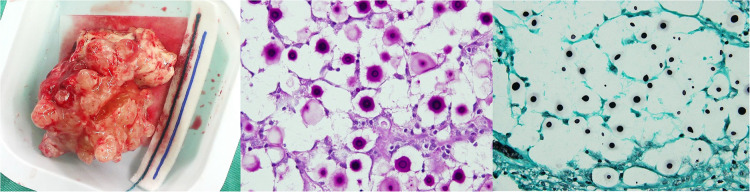
Diffusion-weighted images showed a right frontal mass without water restriction that differed from the abscess (white arrowhead), and a large area of water restriction in the right frontotemporal lobe, suggesting a massive arterial infarction in the MCA vascular region (black arrowhead).

**Figure 3:**
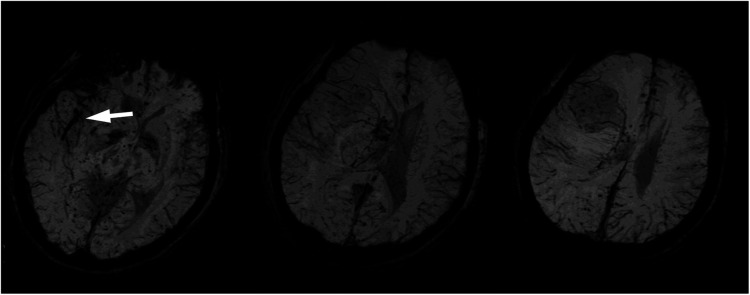
Susceptibility-weighted images showed a mildly increased vascularity in the right frontal mass and a curvilinear dark signal in the right sylvian fissure cistern near the thrombosis of the middle cerebral artery (white arrowhead).

**Figure 4:**
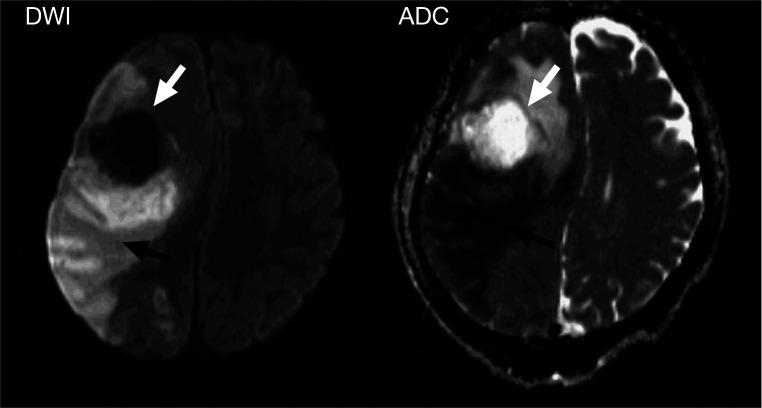
Gross appearance of the resected infectious tumor; microscopic section with PAS; and GMS stain showing hyphal formation.

A 40-year-old male patient presenting with progressive headaches and sudden-onset left-sided weakness was referred to our emergency room. The patient did not have a prior history of any systemic diseases and was otherwise healthy. He worked as a construction worker and denied having any contact with pigeons. At presentation, he was lethargic and febrile, with left-side weakness. Neurologic examination revealed a Glasgow coma scale (GCS) of E3V5M6 with left-sided hemiplegia (muscle power: grade 0), normal cranial nerves, and a positive Babinski sign. Emergency brain computed tomography (CT) was arranged for highly-suspected MCA infarction, and showed an intra-axial mass lesion at the right frontal lobe with severe perifocal edema. A marked midline shift to the left was also noted. A blood test revealed leukocytosis and an elevated C-reactive protein (CRP) level, while chest x-ray showed left lower-lobe consolidation with left paratracheal lymph node enlargement and pleural effusion. Subsequent magnetic resonance imaging (MRI) further delineated a large mass of 53 mm in the right middle frontal lobe with an avid marginal enhancement and central necrosis (Figure 1). Another small lesion of 18 mm in the right caudate head with similar enhancement plus hemorrhaging and diffuse swelling of the right frontal and temporal-occipital lobes was also detected (Figure 2). Right MCA infarction was also apparent on the MRI images (Figure 3). The initial differential diagnoses included a brain abscess, glioma and metastasis. An emergency consultation with a neurosurgeon was conducted under suspicion of a high-grade brain tumor causing cerebral infarct. The patient underwent an emergency craniotomy with en-bloc excision of an elastic solid mass (Figure 4).

After this initial operation, the patient's consciousness became clearer (GCS: E4V5M6); however, the left hemiparesis remained (muscle power grade 1–2). On the 4th day after tumor excision, disturbance of consciousness was again noted, with a GCS of E1V1M5. Emergency brain CT showed deteriorating right hemisphere swelling, a leftward midline shift, obstructive hydrocephalus, and brainstem compression. A frontal lobectomy was performed 6 cm from the anterior frontal tip for decompression. Postoperative recovery was uneventful, except for intermittent episodes of fever that were difficult to control. The pathology report later identified a *Cryptococcus* infection (Figure 4). An infectious disease doctor was consulted, and the patient was started on antifungal treatment with amphotericin B plus flucytosine as per recommendations. After 4 weeks of treatment, the patient recovered well but with continued left hemiparesis. The patient received consolidation therapy with fluconazole (800 mg) for 8 weeks, followed by maintenance therapy with fluconazole (200 mg) for 9 months after discharge. Currently, no recurrence has been noted since completion of the antifungal therapy.

## Discussion

The current case was an intracranial cryptococcoma with ipsilateral MCA infarction in an immunocompetent patient. Due to the uncommon nature of this case, a comprehensive literature review to examine the incidence, clinical manifestations and complications of cryptococcoma was conducted.

*Cryptococcus* meningitis is known to be one of the most common opportunistic infections of the CNS. A review of the literature showed that the incidence of *Cryptococcus* meningitis is approximately one-million people per year, with at least 600,000 mortalities worldwide. Infections most commonly occur in immunosuppressed hosts (1). Another global review study indicated a significant difference in incidence between high-income and middle to low income countries. The 90-day case-fatality rate from HIV-associated *Cryptococcus* meningitis in East Asia, Oceania, Western Europe, and the US is 9%, as compared with 55% in other parts of Asia and South America, and 70% in sub-Saharan Africa. This disparity is due to a combination of earlier access to antiretroviral therapy and the availability of fungicidal drugs in high-income countries (2).

The overall CNS involvement in cryptococcosis was estimated to be 42% in Taiwan (3). The incidence of *Cryptococcus* meningitis ranges from 4.0 to 5.5 per million people per year, with an average incidence of 4.7 per million people per year. A national multicenter epidemiology study in Taiwan from 1997 to 2010 revealed a higher prevalence of cryptococcosis in HIV-negative patients (73%). In addition, 15% of patients had no major comorbidity factors, such as chronic hepatitis or diabetes mellitus. Meningoencephalitis was the most common presentation of cryptococcosis (58.9%) (4).

Compared with these review studies, our case study involved a relatively young male with an HIV-negative status and no underlying conditions such as liver disease, diabetes mellitus, malignancy, kidney disease, or solid organ transplantation. To date, there have only been case reports of cryptococcoma in immunocompetent patients, and no large series studies; therefore, we reviewed the literature to identify these case reports. Table 1 summarizes the case reports from 2013/1/1 to 2016/12/31. To the best of our knowledge, only 15 case reports exist. Headaches, which were the most common symptom, occurred in 10 cases (67%); five patients presented with seizures (33%), four with vomiting (27%), four with a fever (27%), and one with a change in mental status (7%). Muscle weakness was noted in three patients (20%).

**Table 1:** Following list are the reviewed case reports.

No.	Authors and year	Sex/Age	Symptoms	Location	Initial diagnosis	Treatment	Outcome and follow-up
1	Zhu JQ et al., 2013 (5)	F/1	Seizure	Parieto-occipital area	Vascular malformation	Partial Resection and fluconazole	Resolved, 18 years
2	Liu BX et al., 2014 (6)	F/61	Headache, emesis, ataxic gait	Cerebellar	Metastatic tumor or glioma	Surgery	N/A
3	Yeh CH et al., 2014 (7)	M/75	Right side weakness, diminished touch sense	Parietal lobe	Brain tumor	Surgery and fluconazole	Died on 17th day after operation
4	Amburge JW et al., 2016 (8)	M/Mid-aged	Fever, headache, back pain, vomiting, urinary urgency, constipation	Basal ganglion, spine T11∼T12	Cryptococcal meningitis at other hospital	AMB and flucytosine then voriconazole	N/A
5	Dubbioso R et al., 2013 (9)	F/63	Aphasia, confusion	Choroid plexi	Cryptococcoma	AMB and flucytosine then fluconazole	N/A
6	Maciel RA et al., 2016 (10)	M/65	Headache, fatigue, weight loss, left hearing deficit	Cerebral hemispheres, brain stem and cerebellum	Cryptococcus infection	AMB and flucytosine then fluconazole	Died in 4∼5 months
7	Hur JH et al., 2015 (11)	M/47	Headache, nausea, vomiting	Pons	N/A	Surgery and AMB or fluconazole	Resolved, NA
8	Luis SHJ et al., 2013 (12)	M/34	Headache, vomiting, blurred vision	Paraventricular involving the right basal ganglia	Cryptococcus infection	AMB and fluconazole	Died in 5 days
9	Kawamuru I et al., 2014 (13)	M/41	Fever, cough, headache	Throughout cerebral parenchyma	Cryptococcoma	AMB and flucytosine then fluconazole	Resolved, NA
10	Asanuma Y et al., 2014 (14)	M/52	Nocturia, perianal pain, right lower limb motor palsy	Sacral spine	N/A	Laminectomy → fluconazole → AMB → itraconazole	Resolved, NA
11	Suchitha S et al., 2012 (15)	M/70	Right thigh painless swelling	Right thigh, chest and parietooccipital lobe	Soft tissue sarcoma	AMB and fluconazole	Resolved, NA
12	Franco-paredes C et al., 2015 (16)	F/18	Headache, change of mental status, seizure, fever	Bilateral basal ganglia and head of the caudate lobes	Fungal encephalitis	AMB and flucytosine	Died approximately 48 h after admission
13	Hagan JE et al., 2014 (17)	F/25	Right paresthesia, muscle weakness, headache	Thalamus	Glioblastoma multiforme	AMB then fluconazole	Mild sequelae, 4 years
14	Batista R.R. et al., 2014 (18)	M/37	Headache, seizure	Fronto-parietal area	N/A	AMB	N/A
15	Dusak A et al., 2011 (19)	M/33	Headache, fever, seizure, visual field defects	Ventricle and basal ganglion	N/A	N/A	N/A

It was generally known that cryptococcomas can present as a single lesion or multiple lesions and occur in the cerebral parenchyma, basal gangalion, cerebellum, pons, and choroid plexus. In our literature review, there were 6 cases of cryptococcoma occurring in the cerebral parenchyma (40%) and 3 cases in the basal ganglion (20%) (Table 1). However, no case presented with MCA infarction according to the review, which implies that the current case had a particularly rare presentation with an unusual large MCA infarction.

In CNS fungal infections, acute cerebrovascular events take the form of either ischemic (commonly) or hemorrhagic (uncommonly) strokes. Aspergillus, Zygomycetes, Candida, Coccidioides, Histoplasma, Cryptococcus, Penicillium, etc. are all known fungal infections rarely presenting with acute cerebrovascular events (20). R. Raman et al. stated the hypothesis that gradual contiguous involvement of the skull base structures in cases of prolonged paranasal fungal sinusitis (commonly aspergillosis, zygomycosis, cladosporiosis, etc.) leads to angio-invasion, which in turn results in fungal vasculitis, and thereafter thrombotic occlusions occur in the major branches of the cerebral vasculature at the skull base: internal carotid arteries and/or vertebro-basilar system (20). In the current case, our hypothesis was that hyphae invaded the vessel walls of major vasculature at the skull base and caused cerebral arterial thrombosis, cerebral infarction and cerebritis. Sharma et.al stated that in a series of 170 patients, there were 45 cases of major cerebral artery thrombosis, especially either in the ICA or the basilar artery. Ischemic cerebral strokes also result due to cardiac emboli in patients with fungal endocarditis.

Diagnosis of CNS cryptococcoma remains difficult. According to our review, 4/15 (27%) patients were misdiagnosed and treated for brain tumors or a vascular malformation until pathologic examination confirmed a *Cryptococcus* infection. In the review of Li, Q et al., the rate of misdiagnosis was 9/17 (53%) (21). The reasons for frequent misdiagnosis are described below.

First, symptoms are nonspecific. The common symptoms identified in our review, which included headaches, seizures and fever, are often seen in other CNS diseases. The common initial signs of cryptococcomas are an increasing intracranial pressure (IICP) and neurological focal signs (6). These symptoms are also present in brain tumors or abscesses. The IICP sign leads to a poor prognosis and makes diagnosis more difficult. Typically, *Cryptococcus* infections can be detected by CSF antigen titer and CSF analysis *via* lumbar puncture, which can be less invasive than a surgical biopsy. In our case, lumbar puncture was not possible due to the contraindication of IICP.

Second, it is difficult to diagnose cryptococcoma on the basis of imaging. However, according to some of the reports in the literature, the imaging characteristics of CNS cryptococcoma could also be seen in granulomatous tumors and abscesses (6). The common findings in images of brain cryptococcoma were multifocal basal ganglia and midbrain T2 hyperintensities due to gelatinous pseudocysts (cryptococcomas) and dilated Virchow-Robin spaces (22, 23). In our case, the small lesion in the caudate head also represented a typical image-based finding. Brain infarction complicated by infection is commonly seen in aspergillosis, but not in cryptococcosis (24, 25). Multiple lesions with infarctions or hemorrhaging are of a random distribution due to the angio-invasive nature. Hemorrhaging occurs in approximately 25% of lesions (25).

Third, brain abscesses arising from *Cryptococcus* infections are considered rare in immunocompetent patients. The incidence of other intracranial lesions is higher than those arising from *Cryptococcus* infection. However, a review of local studies revealed that *Cryptococcus* meningitis in HIV-negative patients is not uncommon in Taiwan (73% without HIV infection) (4), and that *Cryptococcus neoformans* is the most frequent species in CNS cryptococcosis (1). A genotype study by Tseng, H.K., et al. showed that *C. neoformans* is the most prevalent species of *Cryptococcus* in Taiwan (4). In addition, the variant *Cryptococcus gattii* often infects immunocompetent patients and grows mainly in the soil under Eucalyptus trees; it is not present in avian feces (16).

In our case, the patient was a non-HIV-infected, non-transplant host, but presented with cryptococcoma and not meningoencephalitis. For the treatment of a non-HIV-infected, non-transplant host with complications, we followed the practical guidelines of the Infectious Disease Society of America (26): induction therapy administered as amphotericin B (0.7–1 mg/kg per day I.V.), liposomal amphotericin B (3–4 mg/kg per day I.V.), or amphotericin B lipid complex (5 mg/kg per day I.V.) plus flucytosine (100 mg/kg per day orally in 4 divided doses) for at least 6 weeks. Consolidation and maintenance therapy was completed with fluconazole (400–800 mg per day orally) for 6–18 months. The guidelines also suggest surgery for large (≥3-cm) or accessible lesions with mass effects. In our case, the large lesion measured 5.3 cm with a mass effect; thus, surgical intervention was recommended.

## Conclusion

Diagnosing fungal infections, especially *Cryptococcus* CNS infections, that present as a space-occupying lesion in an immunocompetent patient remains challenging according to the unspecific symptoms. Intracranial cryptococcoma can lead to ischemic cerebral stroke, and can mimic a brain tumor, and therefore *Cryptococcus* infection should always be considered in patients with brain mass lesions with abnormal lab data.

## Data Availability

The raw data supporting the conclusions of this article will be made available by the authors, without undue reservation.
